# Differences in metabolic rate between two Atlantic cod (*Gadus morhua*) populations estimated with carbon isotopic composition in otoliths

**DOI:** 10.1371/journal.pone.0248711

**Published:** 2021-04-01

**Authors:** Szymon Smoliński, Côme Denechaud, Gotje von Leesen, Audrey J. Geffen, Peter Grønkjær, Jane A. Godiksen, Steven E. Campana

**Affiliations:** 1 Institute of Marine Research, Nordnes, Bergen, Norway; 2 Department of Fisheries Resources, National Marine Fisheries Research Institute, Gdynia, Poland; 3 Department of Biological Sciences, University of Bergen, Bergen, Norway; 4 Faculty of Life and Environmental Sciences, University of Iceland, Reykjavik, Iceland; 5 Aquatic Biology, Department of Bioscience, Aarhus University, Aarhus, Denmark; Union College, UNITED STATES

## Abstract

The isotopic composition of inorganic carbon in otoliths (*δ*^*13*^*C*_*oto*_) can be a useful tracer of metabolic rates and a method to study ecophysiology in wild fish. We evaluated environmental and physiological sources of *δ*^*13*^*C*_*oto*_ variation in Icelandic and Northeast Arctic (NEA) cod (*Gadus morhua*) over the years 1914–2013. Individual annual growth increments of otoliths formed at age 3 and 8 were micromilled and measured by isotope-ratio mass spectrometry. Simultaneously, all annual increment widths of the otoliths were measured providing a proxy of fish somatic growth. We hypothesized that changes in the physiological state of the organism, reflected by the isotopic composition of otoliths, can affect the growth rate. Using univariate and multivariate mixed-effects models we estimated conditional correlations between carbon isotopic composition and growth of fish at different levels (within individuals, between individuals, and between years), controlling for intrinsic and extrinsic effects on both otolith measurements. *δ*^*13*^*C*_*oto*_ was correlated with growth within individuals and between years, which was attributed to the intrinsic effects (fish age or total length). There was no significant correlation between *δ*^*13*^*C*_*oto*_ and growth between individuals, which suggests that caution is needed when interpreting *δ*^*13*^*C*_*oto*_ signals. We found a significant decrease in *δ*^*13*^*C*_*oto*_ through the century which was explained by the oceanic Suess effect-admixture of isotopically light carbon from fossil fuel. We calculated the proportion of the respired carbon in otolith carbonate (*C*_*resp*_) using carbon isotopic composition in diet and dissolved inorganic carbon of the seawater. This approach allowed us to correct the values for each stock in relation to these two environmental baselines. *C*_*resp*_ was on average 0.275 and 0.295 in Icelandic and NEA stock, respectively. Our results provide an insight into the physiological basis for differences in growth characteristics between these two cod stocks, and how that may vary over time.

## Introduction

Otoliths are calcium carbonate structures forming part of the acoustic-lateralis system in fish. They grow by accretion of new material across the outer surface, a continuous process throughout the life of the individual. Seasonal changes in the growth rate of fish induce the formation of translucent and opaque zones in the otolith, which together typically form annual increments. The incremental growth allows for the estimation of fish age and for attribution of chemical signals to specific and discreet time periods (e.g. calendar years or seasons). Otolith growth is strongly correlated with individual somatic growth [1], and the growth record and chemical composition reflect the physiological state of an individual fish during its life and its responses to changes in the environment [2].

Stable carbon isotope ratios in otoliths (*δ*^*13*^*C*_*oto*_) can provide information about the metabolic state of fish [3]. The carbon in otolith aragonite is drawn from dissolved inorganic carbonate (DIC) in the ambient seawater and metabolically derived carbon released to the blood stream from the respiration [4]. Carbon from metabolic sources is significantly ^*13*^*C* depleted (e.g. from -20 to -17‰) when compared to seawater DIC (e.g., from 0 to 2‰). With knowledge of *δ*^*13*^*C* in otoliths and environmental sources, it is possible to estimate *C*_*resp*_—the proportional contribution of the respired (metabolic) carbon in otolith. The relationship between *C*_*resp*_ (estimated to range from 0 to 0.95 [3, 5, 6]) and oxygen consumption has been described for several species, so *C*_*resp*_ is increasingly used as a proxy of field metabolic rate, an important measure of physiological performance in free-ranging organisms. Oxygen consumption or metabolic rate are challenging to measure in field conditions, particularly in aquatic environments. Most of the traditional methods are dedicated to laboratory-based research and their adoption for free-swimming fish is logistically difficult [7]. Thus, the proxy provided by *δ*^*13*^*C*_*oto*_ is a valuable tool for ecophysiology [8, 9].

Field metabolic rate is the sum of three components: standard metabolic rate (minimum metabolic rate needed to sustain life at a specified temperature), specific dynamic action (associated with the cost of processing food), and activity metabolism (associated e.g. with swimming, feeding, etc.) [8]. Estimates of field metabolic rate, calculated as *C*_*resp*_, combined with the estimates of assimilated energy, may provide information on the amount of energy available for growth and reproduction—two biological processes which have a profound influence on population resilience [8, 10]. Thus, *C*_*resp*_ as a proxy of field metabolic rates can be used for the investigation of how fish grow and reproduce under different environmental conditions [9]. It provides a unique method to study physiological ecology in fish at the individual level and across the whole lifetime [3]. Moreover, considering the availability of historical otoliths in archives worldwide, time series of *C*_*resp*_ calculated based on *δ*^*13*^*C*_*oto*_ can provide a unique opportunity for long-term retrospective assessment of the physiological performance of fish [3, 8] and changes in the important attributes, e.g. diet variability or environmental tolerance [11].

*δ*^*13*^*C*_*oto*_ is affected both by intrinsic and extrinsic processes [12], which can complicate its interpretation and the estimation of field metabolism. Age and growth rate, as well as temperature, trophic position, and depth distribution, are among the factors that have been shown to influence *δ*^*13*^*C*_*oto*_ through the changes in the metabolic activity of fish, or *δ*^*13*^*C* values in fish diet and seawater DIC [13]. The contribution of the intrinsic and extrinsic factors needs to be evaluated in order to interpret *δ*^*13*^*C*_*oto*_ changes and to use *C*_*resp*_ as a metabolic proxy [3]. Studies of the chemical composition of otoliths that simultaneously control for environmental (e.g. temperature) or physiological effects (e.g. changes of the growth rate of individual fish) help to disentangle different sources of variation and improve the ecological interpretation of chemical composition of otoliths [14–16].

**Fig 1:**
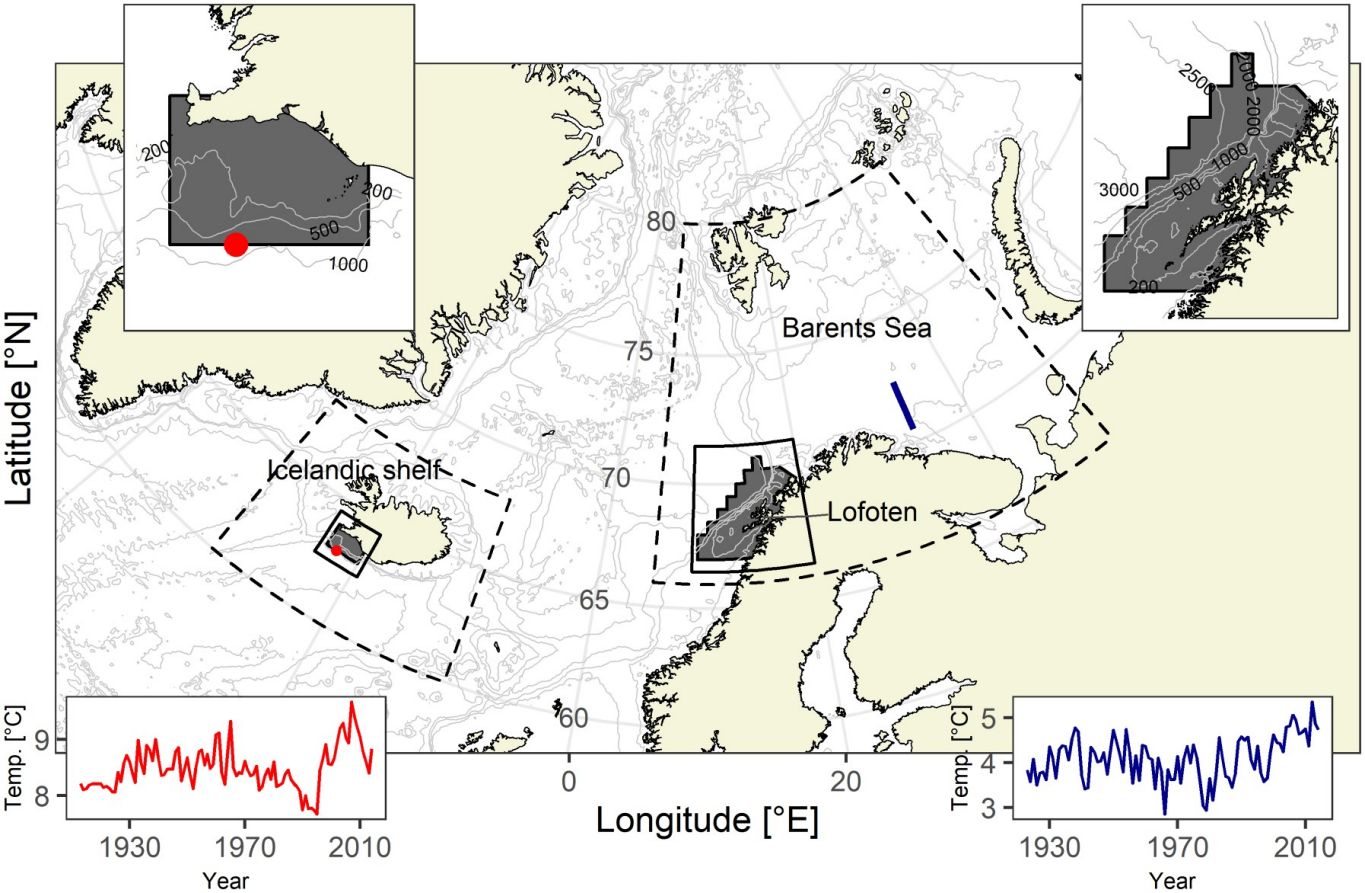
Icelandic and Northeast Arctic cod (*Gadus morhua*) distribution areas. Sampling regions (dark grey polygons) and isobaths (the light grey lines) are marked. The bounding box of the enlarged map is indicated with a solid polygon. The dashed polygons show the areas from which predicted *δ*^*13*^*C*_*DIC*_ values were gathered. Annual mean temperatures at depth for each location, extracted respectively from the selected station (red dot) and the Kola section (solid blue line), are presented at the bottom corners. The map was created based on the bathymetric [21] and shoreline [22] data.

In this study, we measured the carbon isotopic composition of Icelandic and Northeast Arctic (NEA) cod (*Gadus morhua*) otoliths over a century time scale (1914–2013) and analyzed the influence of intrinsic and extrinsic factors on *δ*^*13*^*C*_*oto*_. Icelandic and NEA cod have ranked among the largest stocks of Atlantic cod in the world and are important components of the marine ecosystem [17]. Icelandic cod occur primarily on the Icelandic shelf and spawn around the island [18], but their main spawning ground is located off southwest Iceland [19]. NEA cod inhabit the Barents Sea and spawn mainly around the Lofoten archipelago along the northwest coast of Norway [20] (Fig 1). The utilization of growth and otolith isotopic data from two different, well-characterized, stocks enabled us to test a wider range of environmental conditions across a significant time period.

The aims of this study were i) to evaluate environmental and physiological sources of variation in *δ*^*13*^*C*_*oto*_; ii) to investigate the relationship between *δ*^*13*^*C*_*oto*_ and fish growth; iii) to estimate *C*_*resp*_ as a metabolic proxy based on *δ*^*13*^*C*_*oto*_. We used univariate and multivariate mixed-effects models and estimated conditional correlations between carbon isotopic composition and growth of fish controlling for different intrinsic and extrinsic effects on both otolith traits (*δ*^*13*^*C*_*oto*_ and increment width). We used a stable isotope mixing model to estimate the mean proportion of metabolically derived carbon in otolith carbonate—a metabolic proxy that is corrected for the differences between stocks in the isotopic composition of diet and environment. Our results provide an insight into the physiological basis for differences in growth characteristics between these two cod stocks, and how that may vary over time.

## Materials and methods

### Otolith sampling and processing

Otoliths of Icelandic and NEA cod were collected from the archives of the Marine and Freshwater Research Institute in Hafnarfjörður, Iceland, and the Institute of Marine Research in Bergen, Norway. All otoliths came from the sampling of the commercial catches and from scientific surveys in the spawning areas in southwest Iceland (1929–2015) and around the Lofoten archipelago (1933–2015) (Fig 1). Using archival information on fish age at capture, we aimed at a random collection of 3 individuals caught at age 10+ per sampling year for each stock (S1 Table of S1 File). Information about the location of catch (detailed geographical coordinates or fishing area), date of catch, and biological parameters (total length and sex) were available for most of the individuals. In total, otoliths of 436 fish (213 from Icelandic and 223 from NEA stock) were collected for the isotopic analysis, representing fish life history over a century time scale (1914–2013).

**Fig 2:**
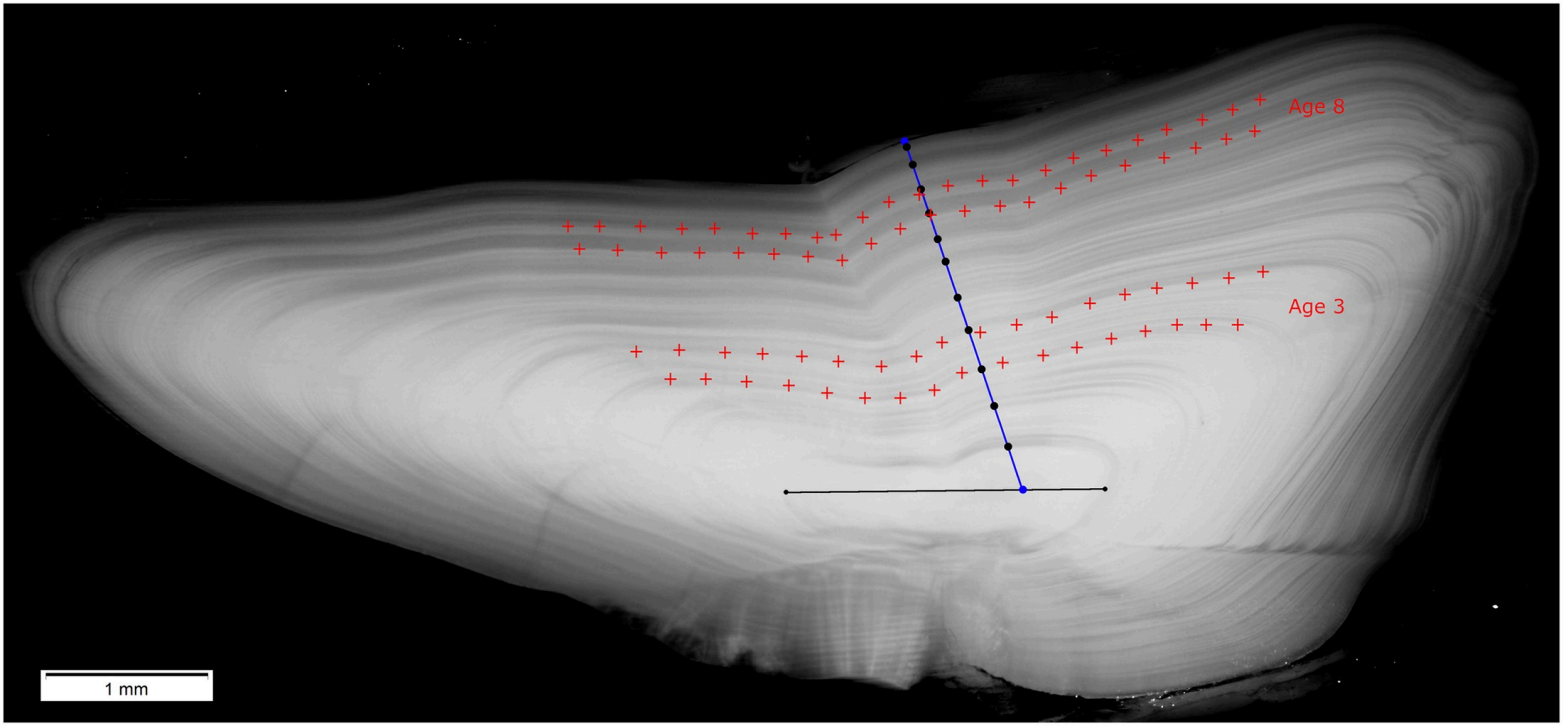
Example of a cod otolith section showing the increment measurement axis (blue) and milled regions (growth increments formed at age 3 and 8 are located between red crosses).

The otoliths were embedded in epoxy resin and ~1 mm-thick transverse sections were cut through the core to reveal the concentric layers of the annual growth increments. Otolith sections were photographed, and the width of each annual increment was measured from the core to the outer edge along the distal axis following a standard protocol (Fig 2). Age was estimated by one reader for each stock in order to maintain consistency and these estimates (not archival) were used throughout analysis. Each increment was assigned to the year of formation by counting back from the known date of capture and accounting for marginal increment interpretation. Additionally, the majority of otolith increments from both stocks were visually assessed and designated as “spawning zones” (the distinctive increments which are believed to be formed after the onset of sexual maturation) using standard procedures for NEA cod age reading [23, 24]. The first and last increment widths were excluded from further analysis since they may not reflect a whole year of growth. Further details on the otolith sampling, processing, and measurement can be found in [25, 26].

Carbonate powder was milled from individual annual growth increments formed at age 3 and age 8 (Fig 2). In a few cases it was not possible to mill these increments and samples from increments formed at age 2 (0.2% of samples) or age 7 (3.5% of samples) were collected (S2 Table of S1 File). Typically, two carbonate samples were obtained from each otolith. Otoliths were milled with high precision using a computer-controlled Merchantek New Wave MicroMill equipped with a digital camera. Translucent and opaque growth zones were combined in one sample to provide a pooled isotope record for the entire year. Samples were drilled up to 750 μm depth (20–25 passes with a drill depth of 30 μm/pass) to obtain approximately 50 μg of otolith carbonate powder in each sample. Further details on the carbonate powder sampling procedure can be found in [27].

Analysis of carbon isotope ratios was conducted on a MAT 253 mass spectrometer at the Institute of Geosciences, University of Bergen, Norway. Carbon isotopic ratios were reported as in parts per thousand or per mil (‰) units with respect to the Vienna Pee Dee Belemnite (VPDB) scale using NBS-19 (*δ*^*13*^*C* = 1.95‰) and NBS-18 (*δ*^*13*^*C* = -5.01‰) standards [28]

$$\delta^{13}C=[(\frac{R_{sample}}{R_{standard}})-1]\times 10^{3},$$

where *R* is the ^*13*^*C/*^*12*^*C*. The long-term reproducibility (1σ precision) of the equipment was *δ*^*13*^*C* ≤0.4‰ for sample sizes greater than 13 μg based on replicate measurements of an internal carbonate standard over a period of months. Data were not corrected for the aragonite acid fractionation.

### Temperature data

Annual mean sea temperatures for the Icelandic spawning ground were extracted from Hadley Centre EN4.2.1 dataset for the location 63°00’ N 22°00’ W (Fig 1) over the depths 0–200 m [29]. The subsurface temperature in this area was assumed to be a proxy of the thermal conditions experienced by the Icelandic cod. For NEA cod annual mean sea temperature data was obtained from the stations 3–7 of the Kola section (from 70°30’ N 33°30’ E to 72°30’ N 33°30’ E, Fig 1) over the depths 0–200 m [30–32]. NEA cod are resident year-round in the Barents Sea as juveniles, and once mature they only leave for their annual spawning migration lasting a few months. Since most of the temperature variations in the Barents Sea and Lofoten spawning area are driven by the same large-scale climatic factors associated with Atlantic water masses, the Kola section is a good representation of the climatological temperatures within the area occupied by the NEA cod throughout their whole life [20, 33, 34]. Both temperature time series covered the entire period represented by the otolith growth and isotopic composition measurements.

### Modeling of otolith traits (*δ*^*13*^*C*_*oto*_ and increment width)

#### Evaluation of environmental and physiological sources of variation in *δ*^*13*^*C*_*oto*_ and growth

A linear mixed-effects modeling framework [35, 36] was applied in order to take into account repeated measurements (both *δ*^*13*^*C*_*oto*_ and width of otoliths growth increment) from the same individual or year of formation (Table 1). This method allows for the partitioning of the variance observed in the traits of cod otoliths with consideration of the intrinsic and extrinsic sources of variation [37, 38]. Two separate univariate mixed-effects models were developed, with either *δ*^*13*^*C*_*oto*_ (representing metabolic processes) or increment width (representing somatic growth) as the response variable. Stock, Age, Sex, and body total length (TL, in cm) were treated as intrinsic effects in both models. The Age term was included in the *δ*^*13*^*C*_*oto*_ model as a fixed factor with two levels (3, 8), while in the increment width model Age was included as a continuous covariate. Sex was included in both models as a fixed factor with males, females, and individuals of unknown sex (lack of archival information) as separate groups. The TL term was incorporated in order to correct model estimates and test the potential effects of certain phenotypes (e.g., fish with larger body size at age). We allowed for the interaction between TL and Stock, as well as three-way interaction between Age, Sex, and Stock. The characterization of increments as “spawning zones” was included with the binary term (SZ: yes/no). Only relatively simple random structures, containing random intercepts for individual fish (FishID) and year of otolith increment formation estimated for each stock (StockYear), were tested in the model due to the limited number of observations. This allowed us to assess the magnitude of the variation associated with between-individual and between-year differences [39]. Increment width and Age were log-transformed before the analysis to meet model assumptions of normality and homogeneity of variance. Fixed covariates (Age, TL, AnomT, Year) were mean-centered in order to facilitate model convergence [37].

**Table 1:** General workflow and variables used in the consecutive steps of the statistical analysis.

Step	Aim	Modeling framework	Response variable	Random structure	Fixed intrinsic effects	Fixed extrinsic effects	Mix sources
1.	Evaluation of environmental and physiological sources of variation in *δ*^*13*^*C*_*oto*_ and growth	Univariate models	*δ*^*13*^*C*_*oto*_ **or** otolith increment width	Fish ID, StockYear	Stock, Sex, TL, Age, SZ	AnomT, Year	
2.	Investigation of the relationship between *δ*^*13*^*C*_*oto*_ and growth	Bivariate model	*δ*^*13*^*C*_*oto*_ **and** otolith increment width	Fish ID, StockYear	Stock, Sex, TL, Age	AnomT, Year	
3.	Estimation of *C*_*resp*_ as a metabolic proxy	Stable isotope mixing model	*δ*^*13*^*C*_*oto*_	Fish ID, StockYear			*δ*^*13*^*C*_*diet*_, *δ*^*13*^*C*_*DIC*_

Fish ID—unique identifier of the fish individual; StockYear—stock-specific effect of the year; TL—total body length of fish; SZ—otolith “spawning zone” (included only in the univariate modeling of *δ*^*13*^*C*_*oto*_); AnomT- temperature anomaly for the given area.

Mixed-effects models with different levels of complexity were compared using the Akaike Information Criterion corrected for small sample sizes (AICc) to select the best base model describing variation in otolith traits. The optimal random structure was selected by comparison of models fitted using restricted maximum likelihood (REML) and the most complex fixed structures [37, 38]. The optimal fixed effects were selected by comparison of models fitted using maximum likelihood and previously identified optimal random structures. In an additional analysis, the SZ term was added to the optimal intrinsic model to test for differences in *δ*^*13*^*C*_*oto*_ between increments assigned as “spawning zones” or not to test for metabolic signals associated with reproduction. Further, the same optimal intrinsic model was extended by using the anomaly of annual mean temperature calculated separately for each stock (AnomT; based on the data gathered from the profile in SW Iceland and the Kola section). We allowed for the interaction between AnomT and the stock term to test possible stock-specific effects of temperature on otolith traits. Additionally, a continuous Year effect was added to allow for the interaction with the stock term, in order to test for long-term stock-specific trends in the otolith traits. Alternative models were again compared using AICc and the best-ranked model was then refitted with REML, allowing unbiased parameter estimates [35]. Assumptions of the final models were checked and satisfied with standard diagnostics. The significance of fixed effects was assessed based on conditional F-tests with Kenward-Roger approximation for the degrees of freedom [40]. Predicted effects of the variables selected during comparisons of AICc were estimated and visualized. The intraclass correlation coefficient (ICC) was calculated in order to assess between-individual and between-year differences in otolith traits [41, 42]. The conditional and marginal R^2^ metrics were calculated for both models to assess the amount of variance in otolith traits explained by random effects alone and both fixed and random effects, respectively [43].

#### Investigation of the relationship between *δ*^*13*^*C*_*oto*_ and growth

The variables considered in the analysis (*δ*^*13*^*C*_*oto*_ and growth increment width) represent repeated measures of different otolith traits that are not independent of one another [14]. Therefore, bivariate linear mixed-effects models [44] with both measured otolith traits as response variables were developed. Bivariate linear mixed-effects models allow for the simultaneous estimation of the variance of each response and the covariance between them, at group levels specified within the random effects structure [45]. Potential correlations of otolith traits can be decomposed into within-individual, between-individual, and between-year correlations through the partitioning of the variance at different levels of random effects [46]. Importantly, correlation estimates between otolith traits obtained with multivariate mixed-effects models are unbiased and derived with adequate quantification of uncertainty in specified random effects [47].

All bivariate linear mixed-effects models were fitted with the optimal random and fixed effects identified during the development of univariate models [14]. Series of bivariate models were built in order to attribute potential covariances to the set of intrinsic or combined intrinsic and extrinsic fixed effects [46]. Therefore, correlations between increment widths and *δ*^*13*^*C*_*oto*_ were tested within the bivariate framework which included i) random effects only, ii) random effects and intrinsic fixed effects, and iii) random effects, intrinsic and extrinsic fixed effects.

Measurements of increment width were available for all years of fish life, while *δ*^*13*^*C* in the otolith carbonate was measured only from annual growth increments formed at age 3 and 8. However, multivariate linear mixed-effects models are able to deal with missing values for response variables [44]. The bivariate model with both otolith traits as response variables was fitted to the data using a Bayesian approach and Markov Chain Monte Carlo methods [44]. Prior to the fitting, response variables were standardized to ensure similar scale, and growth increment measurements were log-transformed to meet the assumptions of multivariate normality [14]. The estimates of parameters were evaluated based on the model run with parameter-expanded priors for 120,000 iterations with a burn-in phase of 20,000 and a thinning interval of 10. Model assumptions were checked by visual inspection of residuals and by analysis of autocorrelation of the chains. The resulting matrices of within-individual, between-individual, and between-year (co)variance of both otolith traits were used to calculate point estimates and 95% credible intervals, which were further compared between models with different effects incorporated. Correlations where credible intervals did not overlap zero were considered significant.

#### Estimation of *C*_*resp*_ as a metabolic proxy

Long-term trends in biogenic carbonate *δ*^*13*^*C* measurements are subject to mis-interpretation due to the Suess effect, i.e. decrease of *δ*^*13*^*C*_*DIC*_ in the seawater due to the penetration of isotopically light fossil fuel CO_2_ into the oceans [48]. Therefore, following recommendations in previous historical and paleoclimate studies [3], the *δ*^*13*^*C*_*oto*_ values were corrected prior to the estimation of *C*_*resp*_ with the Bayesian stable isotope mixing model using a Year slope estimated during univariate modeling.

The mean proportion of respiratory carbon in otolith carbonate (*C*_*resp*_) was estimated through a Bayesian stable isotope mixing model [49], using a two-source input mass balance equation [50]:

$$\delta^{13}C_{oto}=C_{resp}*\delta^{13}C_{diet}+(1-C_{resp})*\delta^{13}C_{DIC}+\varepsilon ,$$

where *δ*^*13*^*C*_*oto*_ is the corrected *δ*^*13*^*C* measured in the otolith increments, while *δ*^*13*^*C*_*diet*_ and *δ*^*13*^*C*_*DIC*_ are the average *δ*^*13*^*C* values of the diet and DIC in seawater. Since *C*_*resp*_ represents proportion, it is a unitless quantity. The *ε* term is the total net isotopic fractionation during carbon exchange (between DIC and blood and between blood and endolymph in which the otolith is formed), which was set to 2.7‰ [13, 51]. The values of *δ*^*13*^*C*_*DIC*_ within the depth range occupied by cod in each study area [52–56] were estimated based on the apparent oxygen utilization values [57, 58] obtained from the Global Ocean Data Analysis Project version 2—GLODAPv2 database [59] (see S1 File for the detailed description). The values of *δ*^*13*^*C*_*diet*_ were approximated based on the published information [60–64] (see S1 File for the detailed description).

Bayesian models simultaneously consider isotopic variations of both sources (*δ*^*13*^*C*_*DIC*_ and *δ*^*13*^*C*_*diet*_) and their mixture (*δ*^*13*^*C*_*oto*_) and allow for the proper inclusion of uncertainty [65, 66]. *C*_*resp*_ was estimated separately for the Icelandic and NEA fish, taking into account the hierarchical structure of the data and repeated measurements by including FishID and Year as random effects. The models were run with uninformative priors for 100,000 iterations with a burn-in phase of 50,000 and a thinning interval of 50. Standard Gelman-Rubin and Geweke diagnostics were used for the evaluation of models’ performance.

All analyses were conducted using the R scientific computing language [67] and the following packages: *lme4* [68], *MCMCglmm* [44], *MixSIAR* [49], *MuMIN* [69].

## Results

### Sources of variation in *δ*^*13*^*C*_*oto*_

**Fig 3:**
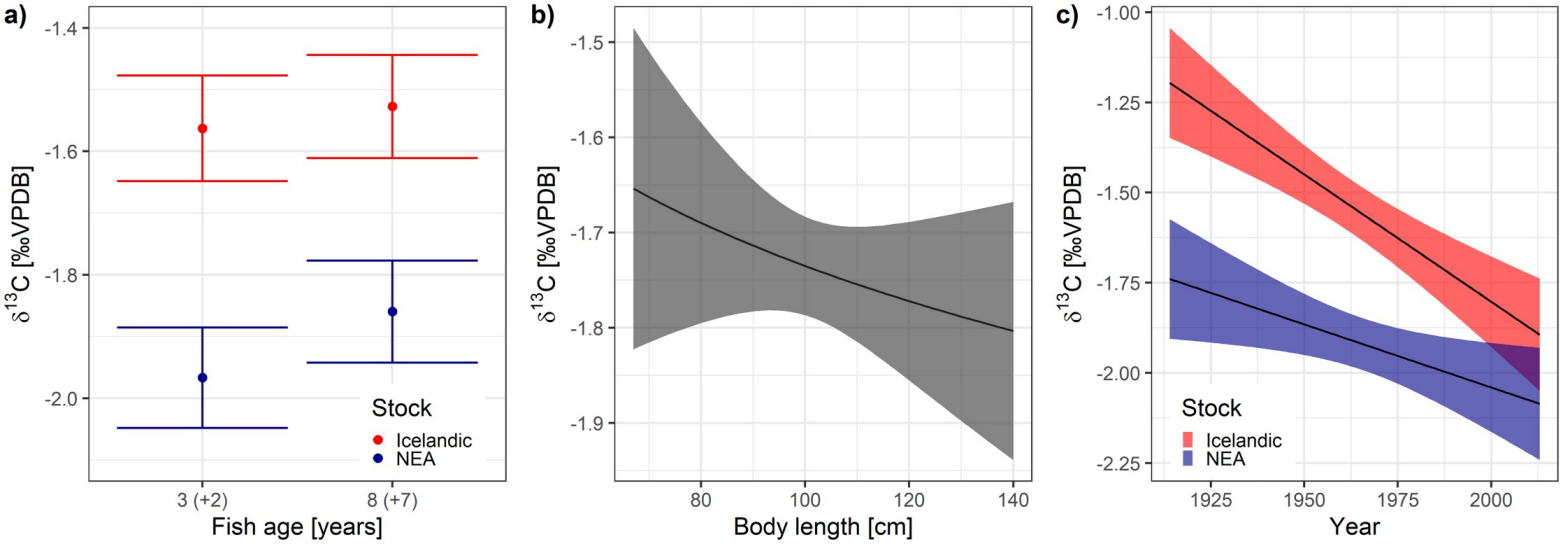
Effects of the variables predicted by the univariate model of carbon isotope ratios selected based on the AICc. The estimated Year slopes (c) which were explained by the oceanic Suess effect were used to correct the *δ*^*13*^*C*_*oto*_ values prior to the estimation of *C*_*resp*_.

A univariate model was developed to identify intrinsic and extrinsic effects on *δ*^*13*^*C*_*oto*_ (see S1 File for the detailed results of the model selection procedure), which were later used to estimate the correlations between *δ*^*13*^*C*_*oto*_ and growth. Otoliths of Icelandic cod had higher *δ*^*13*^*C*_*oto*_ than those of NEA cod (Stock effect p<0.001). Fish at age 8 showed higher *δ*^*13*^*C*_*oto*_ values than fish at age 3 in both stocks, but the differences were not statistically significant (main Age effect p = 0.409), nor was there a significant sex-related effect (term was excluded during model selection based on AICc). *δ*^*13*^*C*_*oto*_ decreased with TL at capture, but the effect was not statistically significant (p = 0.306). Overall, there was a decreasing trend in *δ*^*13*^*C*_*oto*_ over the last century (main Year effect p<0.001), with a larger decrease observed in Icelandic cod (mean values of *δ*^*13*^*C*_*oto*_ decreased by 0.7‰ over the past 100 years) compared to NEA cod (mean values decreased by 0.3‰ over the past 100 years) (Table 2, Fig 3). There was no significant difference in *δ*^*13*^*C*_*oto*_ between “spawning zones” and normal increments (term was excluded during model selection). Temperature did not have a significant effect on *δ*^*13*^*C*_*oto*_ (AnomT term was excluded during model selection). Both FishID (ICC = 0.418), and the Year random effects (ICC = 0.111) explained a significant portion of the variance. Overall, random effects (FishID and StockYear) explained the majority (conditional R^2^ = 0.61), while fixed effects explained only a small fraction of *δ*^*13*^*C*_*oto*_ variance (marginal R^2^ = 0.17) (Table 2).

**Table 2:** Parameter estimates of the optimal univariate models for otolith carbon isotope ratios and increment width selected with AICc. Estimates are given for all fixed effects with confidence intervals (*CI*) and significance (*p*). For the random effects residual variance (*σ*^*2*^), the variance associated with tested effects (*τ*_*00*_) and their intraclass correlation coefficient (*ICC*) are given. The number of observations used to fit model and the amount of variance explained (*marginal* and *conditional R*^*2*^) are specified.

*Predictors*	δ^13^C_oto_			Increment width		
	*Estimates*	*CI*	*p*	*Estimates*	*CI*	*p*
Intercept	-1.563	-1.648 –-1.477	**<0.001**	5.146	5.130–5.162	**<0.001**
Age (factor)*	0.036	-0.048–0.119	0.409			
Age (factor)*:Stock**	0.071	-0.043–0.186	0.223			
Year	-0.007	-0.010 –-0.004	**<0.001**			
Year:Stock**	0.004	-0.000–0.008	0.080			
TL	-0.203	-0.591–0.185	0.306	0.301	0.216–0.386	**<0.001**
Stock**	-0.404	-0.522 –-0.286	**<0.001**	0.041	0.018–0.063	**<0.001**
AnomT				0.020	-0.004–0.043	0.102
Age (continuous)				-0.649	-0.669 –-0.630	**<0.001**
Age (continuous):Stock**				0.133	0.105–0.161	**<0.001**
**Random Effects**						
σ^2^	0.144			0.058		
τ_00_	0.128 _FishID_			0.005 _FishID_		
	0.034 _StockYear_			0.001 _StockYear_		
ICC	0.418 _FishID_			0.076 _FishID_		
	0.111 _StockYear_			0.021 _StockYear_		
N of measurements	836***			4243		
N of random groups	436 _FishID_			436 _FishID_		
	183 _StockYear_			194 _StockYear_		
Marginal R^2^ / Conditional R^2^	0.166 / 0.608			0.604 / 0.642		

*estimates of the coefficient for increment formed at age 8 in relation to age 3.

**estimates of the coefficient for NEA stock in relation to Icelandic stock.

*** for 36 individuals only 1 measurement of *δ*^*13*^*C* was available.

### Sources of variation in growth

**Fig 4:**
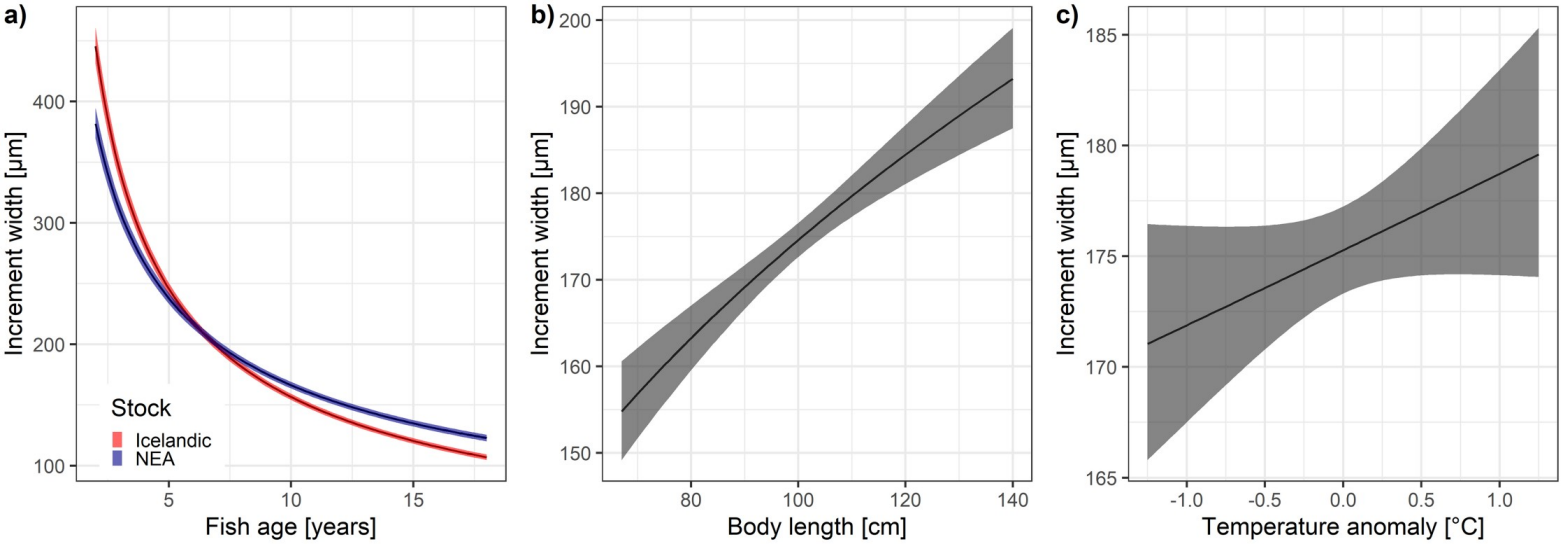
Effects of the variables predicted by the univariate model of otolith increment width selected based on the AICc.

The effects on growth rate were investigated with a univariate model (see S1 File for the detailed results of the model selection procedure). Important intrinsic and extrinsic effects were identified and included in the further step of the analysis in order to estimate correlations between *δ*^*13*^*C*_*oto*_ and fish growth rates conditioned on these effects. Fish growth, as represented by increment width, decreased significantly with age (p<0.001), but there were different age-related growth patterns in the two stocks (Age:Stock interaction p<0.001) (Fig 4a). The Icelandic cod grew faster as young fish, and slower as old fish when compared to NEA cod, but there was no detectable effect of sex (this term was excluded during model selection). No significant long-term linear trends were found in the growth data (Year term was excluded during model selection). There was a positive, albeit not statistically significant, effect of temperature anomalies (AnomT p = 0.102) on growth, which was supported by the model selection based on AICc values (Table 2, Fig 4c). A higher proportion of variance in growth was associated with the random effect of FishID (ICC = 0.076) when compared to random effects of Year (ICC = 0.021), but most of the variance in the growth was explained by the fixed effects (marginal R^2^ = 0.60, conditional R^2^ = 0.64) (Table 2).

### Correlation between *δ*^*13*^*C*_*oto*_ and growth

**Fig 5:**
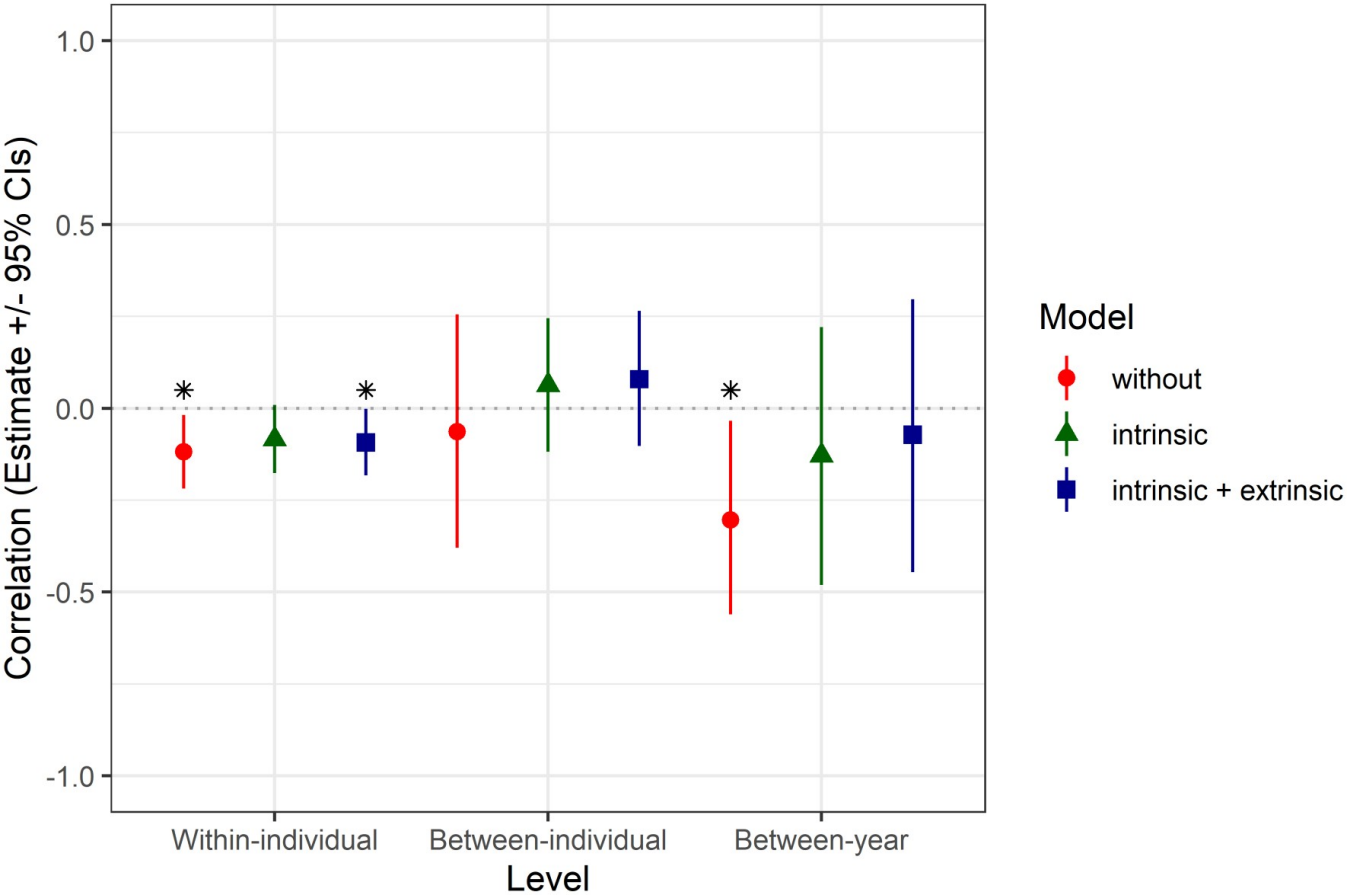
Correlations between *δ*^*13*^*C*_*oto*_ and growth increment width (mean value and 95% credible intervals) estimated within-individual, between-individual, and between-years with the models incorporating different sets of effects (indicated with colors). Significant correlations where credible intervals do not overlap zero are indicated with asterisks.

In the bivariate models *δ*^*13*^*C*_*oto*_ was significantly correlated with growth within-individuals (R = -0.12; 95% credible interval, CI: -0.22 –-0.02) and between-years (R = -0.30; CI: -0.56 –-0.03). This can be attributed to the intrinsic factors since the correlations diminished after incorporation of intrinsic or both intrinsic and extrinsic factors in the bivariate model. There was no significant correlation of *δ*^*13*^*C*_*oto*_ and growth between-individuals (R = -0.06; CI: -0.38–0.26) (Fig 5).

### Estimated *C*_*resp*_

**Fig 6:**
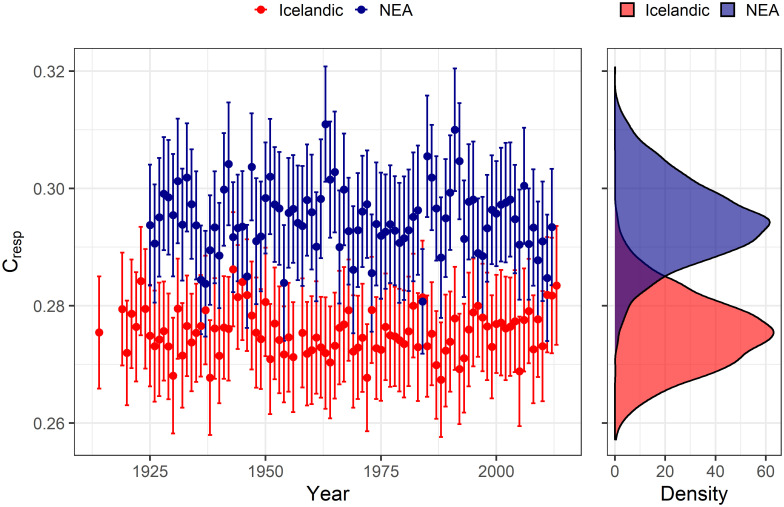
Year-specific (random effect; left panel; mean±sd) and global (right panel) estimates of the proportion of metabolically derived carbon in otolith carbonate (*C*_*resp*_) for Icelandic and NEA cod.

The estimated average *C*_*resp*_ was 0.275 (±0.006) for Icelandic cod and 0.295 (±0.007) for NEA cod and the difference between stocks was significant (95% credible intervals of the estimated difference did not overlap zero). There was noticeable interannual variability in the *C*_*resp*_, but the Year random effect estimates were characterized by the high uncertainty. There was no statistically significant synchrony in the estimated mean annual *C*_*resp*_ between the Icelandic and NEA cod (R = -0.17, p = 0.117) (Fig 6).

## Discussion

In Icelandic cod *δ*^*13*^*C*_*oto*_ was significantly higher than in NEA cod. Differences in the mean *δ*^*13*^*C*_*oto*_ between stocks inhabiting distant areas are expected due to the differences in the oceanographic and ecological conditions, as well as the metabolic activity of the fish [12]. Previous studies, for example in eastern Newfoundland, Canada, indicated that there was a general similarity in *δ*^*13*^*C*_*oto*_ within a more limited geographic area [13]. However, even in this relatively limited geographical space of eastern Newfoundland, different areas were characterized by particular lifetime patterns of *δ*^*13*^*C*_*oto*_ indicating some degree of separation of individuals throughout their lives [13].

Although the *δ*^*13*^*C*_*oto*_ from increments formed at age 3 were generally lower than from increments formed at age 8, the differences among the ontogenetic stages were not statistically significant. Previous studies on Atlantic cod and other species have shown a general increase in *δ*^*13*^*C*_*oto*_ with age [12, 50, 70]. This increase has been attributed to varying fish diet or decreasing size-specific metabolic rate as fish get older, which means a lower contribution of the metabolically derived carbon depleted in ^*13*^*C* [3, 5, 8]. It has been noted that *δ*^*13*^*C*_*oto*_ values increase through the early period of the life of fish, then reach a maximum around the time of sexual maturation, and this period is followed by a stable plateau or decreasing trend as the fish grow older [50]. A decrease in *δ*^*13*^*C*_*oto*_ of mature cod was previously attributed to the migration of the fish to deeper waters characterized by a lower *δ*^*13*^*C* in DIC [13, 50]. It is probable that a large portion of the samples in our study collected from the annual increments formed at age 8 represents a period where the *δ*^*13*^*C*_*oto*_ values had already declined with a changed depth distribution, and therefore, differences between age groups were not highly pronounced.

We also observed a decrease in the *δ*^*13*^*C*_*oto*_ with increasing TL. Intrinsic genetic variation in maximum body size and growth among individuals or life-history types are linked with metabolic rate [71]. Because most of the fish analyzed in this study were caught at age 10 or 11, the TL effect reflects differences in life-long growth rate. The faster growing individuals are characterized by lower *δ*^*13*^*C*_*oto*_, suggesting higher incorporation of metabolically derived carbon (depleted in ^*13*^*C*) in addition to the DIC source during otolith calcification [72]. Therefore, the observed trend supports a relationship between *δ*^*13*^*C*_*oto*_ and the individual’s metabolic rate, which has been reported for different fish species based on field and experimental observations [6, 8, 9].

There were declining trends in *δ*^*13*^*C*_*oto*_ over the last century in both cod stocks. A much stronger decline was observed in Icelandic cod (0.7*‰* over the past 100 years), than for NEA cod (0.3*‰*). We found no significant temporal trends in the otolith growth data, suggesting that the trend in *δ*^*13*^*C*_*oto*_ was associated with effects other than long-term changes in growth rate. The observed trends in *δ*^*13*^*C*_*oto*_ were consistent with direct observations and modeling studies showing a continuous decline in *δ*^*13*^*C* in the seawater DIC over the postindustrial period, caused by the penetration of isotopically light fossil fuel CO_2_ into the oceans, referred to as the Suess effect [48]. A strong Suess effect has been observed in well-mixed waters, and the North Atlantic Ocean is among the areas showing the strongest (more than 0.6‰ per century) decrease of *δ*^*13*^*C* in seawater [48]. A similar decrease (0.5‰ per century) can be calculated based on the *δ*^*13*^*C*_*oto*_ values reported for the years 1919–1992 in a previous study on NEA cod otoliths [73]. We mathematically corrected for the Suess effect using estimated linear year trends. Proper calibration of long-term otolith carbon isotopic data concerning postindustrial depletion in seawater ^*13*^*C* is needed before they can be utilized for the estimation of the changes in metabolic rates of fish [3]. Neglecting this effect may seriously hamper the interpretation of the changes in *δ*^*13*^*C*_*oto*_ and lead to an overestimation of the metabolic rates in the most recent periods [74]. Because the decrease in the seawater *δ*^*13*^*C* has a nonlinear form, with a stronger decreasing trend over the last two decades, more precise methods for the correction for the Suess effect than linear approximation should be applied [75].

We found no statistically significant effect of temperature on *δ*^*13*^*C*_*oto*_, but the range of thermal conditions observed in this study may be too small to reveal temperature effects on *δ*^*13*^*C*_*oto*_. We hypothesized that metabolic factors would influence *δ*^*13*^*C*_*oto*_ and thus we would expect that increased temperature, leading to higher metabolic rates and oxygen demands, should be reflected in decreased *δ*^*13*^*C*_*oto*_ [8]. However, previous experimental and field studies have not consistently shown the same correlation patterns between temperature and *δ*^*13*^*C*_*oto*_ [6]. For example, *δ*^*13*^*C*_*oto*_ was not significantly affected by water temperature in laboratory-reared plaice (*Pleuronectes platessa*) [72], a negative correlation was observed in laboratory-reared Atlantic croaker (*Micropogonias undulutus*) larvae [76], a positive correlation was observed in cod populations in the Northeast Atlantic [70], and a negative correlation in various other fish species in the field [6]. These inconsistencies in the observed relationships suggest a more complex set of controls on *δ*^*13*^*C*_*oto*_ variation than temperature alone and apparently reflect a combination of feeding and physiological processes, in addition to temperature [70]. The impact of temperature on *δ*^*13*^*C*_*oto*_ would not be expected to be direct, but rather indirect through the influence on fish growth and metabolism [6, 50].

We controlled for different effects on lifelong growth rate in order to properly estimate correlations between *δ*^*13*^*C*_*oto*_ and fish growth rates and identify the intrinsic or extrinsic factors which control them [14]. Our predictions from univariate models corroborate previous findings. The growth of fish decreased significantly with age, which is a well-recognized effect [38]. The otolith increment growth proxy indicates that Icelandic cod grew faster in their early ages (1–6) compared to the NEA cod, but the pattern was reversed in the older ages (7 and older). Individuals with higher growth rates throughout their whole life have wider otolith increments and we corrected for this effect by the inclusion of TL term, as higher length at age at point of capture identified faster growing fish. We found positive, albeit not significant, effects of temperature on otolith growth, which indirectly indicates a relationship between fish metabolism and otolith growth [2].

Inter-individual variation in *δ*^*13*^*C*_*oto*_ was approximately 4 times higher than interannual variation in *δ*^*13*^*C*_*oto*_. Fish have different personalities expressed as individual behavioral differences, such as aggressiveness or shyness, which can be linked to differences in migration patterns or food consumption [9]. The interpretation of environmental effects can be complicated by different physiological responses and diversity of life histories of individual fish [2]. In our models, a high level of variance associated with the FishID effect, and low sample size, reduce the statistical power to detect environmental effects and estimate population-level changes in *δ*^*13*^*C*_*oto*_ in relation to the environmental variability. Potential differences in the *δ*^*13*^*C*_*oto*_ between fish from the same locations but characterized by certain life-history types would lead to valuable insights into ecophysiological processes over individual’s whole lifetime. In this study, only the migratory NEA cod were analyzed, and their stock identity confirmed by otolith morphology [77], but the Icelandic samples may have included fish of different ecotypes [78].

We found correlations between *δ*^*13*^*C*_*oto*_ and growth within-individuals, which reflect intrinsic processes and the changes in the assimilation of *δ*^*13*^*C* associated with growth rate differences through the ontogeny. However, we found no significant correlations of *δ*^*13*^*C*_*oto*_ and growth between-individuals. Similarly to our results, *δ*^*13*^*C*_*oto*_ was not influenced by growth variability within or across nursery sites of herring in Icelandic waters [79]. However, previous studies showed that *δ*^*13*^*C*_*oto*_ was negatively related to the growth rate of juvenile plaice (*Pleuronectes platessa*) reared at 11 °C and tended to increase with growth at 17 °C [72], while *δ*^*13*^*C*_*oto*_ was negatively related to the growth rate of cod reared at 6 and 10 °C [5]. The absence of between-individual correlation between *δ*^*13*^*C*_*oto*_ and growth and the observed high interindividual variance in *δ*^*13*^*C*_*oto*_ suggest that metabolic effects may be obscured in the field studies by a complex set of intrinsic and extrinsic effects. These effects were not fully controlled here in these wild populations, as compared to experimental studies [5, 72].

Individual fish experience periods of higher and lower metabolic activity and growth through the year [80], yet growth and *δ*^*13*^*C*_*oto*_ in this study were measured at an annual resolution [13]. Due to uneven otolith accretion rates, annual otolith carbonate samples used in our study represent a weighted mean [27]. Therefore, stronger correlations between *δ*^*13*^*C*_*oto*_ and growth may have been obscured. Sampling with higher temporal resolution would be necessary to investigate seasonal changes in *δ*^*13*^*C*_*oto*_ associated with differences in growth [81, 82]. These links between growth and the isotopic composition of otoliths have to be considered in order to properly reconstruct past environmental histories based on the analysis of *δ*^*13*^*C*_*oto*_ [5].

We found a significant negative correlation between *δ*^*13*^*C*_*oto*_ and growth between-years which was attributable to intrinsic factors. Since our otoliths were in general sampled in an age-balanced manner through the study period, we could assign the observed differences in *δ*^*13*^*C*_*oto*_ directly to body size, representing a life-time growth rate. We observed lower *δ*^*13*^*C*_*oto*_ in otolith carbonates deposited in years characterized by more intensive fish growth. These negative correlations indicate depletion of ^*13*^*C* in otoliths at higher respiration rates [5] and corroborate previous findings and model predictions for cod and other fish species [6, 50]. Typically higher metabolism drives higher consumption and growth, however, higher metabolism can exist also where feeding opportunities are poor and growth is lower [83]. In some cases, despite differences in consumption and metabolic rates, similar growth rates can be observed between fish of varying life-history types [84]. Since growth is a complex physiological phenomenon involving the transformation of food into tissue and the transport of e.g. amino acids, proteins, or lipids in the blood, the chemical composition of otoliths appears to be also an important predictor of growth rate [2].

We calculated *C*_*resp*_ using approximated values of *δ*^*13*^*C*_*DIC*_ and *δ*^*13*^*C*_*diet*_, which allowed us to correct our estimates for each stock in relation to these two environmental baselines [3]. We observed a higher mean *C*_*resp*_ for the NEA cod stock relative to the Icelandic stock. NEA cod is known for its intensive migratory behavior [20, 85]. The long-term physiological state of NEA cod associated with its migratory nature (i.e. elevated locomotor activity, respiration rate, and oxygen consumption [86]) may therefore explain its higher metabolic rate and *C*_*resp*_ which was inferred through the analysis of *δ*^*13*^*C*_*oto*_. Our results (*C*_*resp*_ = 0.275 and *C*_*resp*_ = 0.295 for Icelandic and NEA cod, respectively) corroborate well with previous studies. For example, the mean *C*_*resp*_ estimated based on *δ*^*13*^*C*_*oto*_ for cod specimens collected from different locations in the eastern North Atlantic was 0.2 [70]. A *C*_*resp*_ in the range of 0.07–0.43 was reported for cod in the northeastern Scotian Shelf, Atlantic Canada [50]. The proportion of metabolically derived carbon in the otoliths of larvae and early juvenile cod reared in a controlled laboratory experiment was estimated to be 0.28–0.32 [5]. High agreement of the results obtained in these independent studies suggests that carbon isotope composition in fish otoliths reflect the level of aerobic activity and foraging patterns of wild fish [8, 87]. Retrospective analysis of metabolic history can provide information on important lifestyle attributes, e.g. diet variability, environmental tolerance, or population performance [11].

We based our *C*_*resp*_ estimations on i) measured *δ*^*13*^*C*_*oto*_, ii) predicted *δ*^*13*^*C*_*DIC*_ using data on measured *AOU*, iii) *δ*^*13*^*C*_*diet*_ assumed based on the information available in the literature, iv) fractionation factor (*ε* term) adopted from the previous cod otolith studies, and v) mathematical correction for the long-term trend attributed to the oceanic Suess effect. Each element of this methodological approach introduces unavoidable uncertainties to the analysis, but it is the fractionation factor that seems to be the most critical in the estimation of *C*_*resp*_ [81, 88, 89]. The application of different *ε* term values can cause important differences in calculated *C*_*resp*_ values [88]. We applied *ε* = 2.7% in order to make our findings comparable with previous results [5, 13, 80], but further studies are needed to investigate the specific values of fractionation factor among species and minimize the potential bias of *C*_*resp*_ estimations [3].

This study assessed different sources of variation in the carbon isotopic composition of otoliths in the two biggest cod stocks of the Atlantic cod. Our results show high inter-individual variation in the *δ*^*13*^*C*_*oto*_ signals, which make population-level inferences very difficult. We emphasize the need to consider these inter-individual differences in the analysis of *δ*^*13*^*C*_*oto*_ data. Interpretation of *δ*^*13*^*C*_*oto*_ signals remains challenging because of the intrinsic effects that can influence *δ*^*13*^*C*_*oto*_, besides environmental conditions. Nonetheless, the consistent differences between Icelandic and NEA cod in their *δ*^*13*^*C*_*oto*_ and *C*_*resp*_ provide evidence for underlying physiological basis for the well-documented growth differences between these two stocks. Similarly, observed within-individual and between-years correlations of *δ*^*13*^*C*_*oto*_ and growth indicate a link between the metabolic state of fish and the carbon isotopic composition. Analysis of *δ*^*13*^*C*_*oto*_ has the potential to indicate changes in the aerobic activity of wild fish [87], but more detailed knowledge on the relationships between fish metabolism and *δ*^*13*^*C*_*oto*_ is necessary before *δ*^*13*^*C*_*oto*_ and *C*_*resp*_ proxy can be applied to reconstruct the history of the metabolic state of the wild fish populations.

## Supporting information

S1 File (PDF)
