# Supplementary material for: Differences in metabolic rate between two Atlantic cod (Gadus morhua) populations estimated with carbon isotopic composition in otoliths
Source: PLoS One. 2021 Apr 1;16(4):e0248711. doi: 10.1371/journal.pone.0248711 (PMC8016290; doi:10.1371/journal.pone.0248711)
Supplement: S1 File — (PDF) [file pone.0248711.s001.pdf]

S1 File

Differences in metabolic rate between two Atlantic cod (*Gadus morhua*) populations estimated with carbon isotopic composition in otoliths

Szymon Smoliński[1,2,*], Côme Denechaud[1,3], Gotje von Leesen[4], Audrey J Geffen[1,3], Peter Grønkjær[5], Jane A Godiksen[1], Steven E Campana[4]

[1]Institute of Marine Research, P.O. Box 1870 Nordnes, N-5817 Bergen, Norway

[2]Department of Fisheries Resources, National Marine Fisheries Research Institute, Kołłątaja 1, 81-332 Gdynia, Poland

[3]Department of Biological Sciences, University of Bergen, Bergen, Norway

[4]Faculty of Life and Environmental Sciences, University of Iceland, Reykjavik, Iceland

[5]Aquatic Biology, Department of Bioscience, Aarhus University, Denmark

*Corresponding author

E-mail: szymon.smolinski@hi.no (SS)

1. Otolith $\delta^{13}C$ samples

Table S1. Number of fish available for $\delta^{13}C$ analysis by stock and year of capture.

Year of capture	Icelandic	Northeast Arctic	Year of capture	Icelandic	Northeast Arctic	Year of capture	Icelandic	Northeast Arctic
1929	3	NA	1959	NA	3	1989	2	NA
1930	2	NA	1960	3	3	1990	3	3
1931	3	NA	1961	3	3	1991	2	3
1932	3	NA	1962	1	3	1992	3	3
1933	3	3	1963	NA	3	1993	3	3
1934	3	3	1964	2	3	1994	2	3
1935	3	3	1965	3	NA	1995	3	3
1936	3	3	1966	NA	NA	1996	2	3
1937	3	3	1967	2	3	1997	2	3
1938	3	3	1968	3	3	1998	3	3
1939	3	3	1969	3	3	1999	1	3
1940	3	3	1970	2	3	2000	2	3
1941	3	3	1971	3	3	2001	NA	3
1942	3	3	1972	NA	3	2002	1	3
1943	3	3	1973	3	3	2003	3	3
1944	3	3	1974	3	3	2004	2	3
1945	3	3	1975	2	3	2005	2	2
1946	4	3	1976	2	3	2006	3	3
1947	3	3	1977	3	3	2007	3	3
1948	2	3	1978	2	3	2008	3	3
1949	2	3	1979	3	3	2009	3	3
1950	3	3	1980	3	NA	2010	3	3
1951	3	3	1981	2	NA	2011	3	3
1952	3	3	1982	2	3	2012	2	3
1953	NA	3	1983	3	NA	2013	1	3
1954	3	3	1984	NA	NA	2014	3	3
1955	3	3	1985	3	3	2015	3	2
1956	3	3	1986	3	NA			
1957	3	3	1987	3	3			
1958	3	3	1988	3	3			

Table S2. Number of otolith $\delta^{13}C$ samples by stock and age at which otolith increments were formed.

Stock	Age	N
Icelandic	3	192
Icelandic	7	11
Icelandic	8	195
Northeast Arctic	2	1
Northeast Arctic	3	220
Northeast Arctic	7	4
Northeast Arctic	8	213

2. Estimation of $\delta^{13}C$ in DIC

The Global Ocean Data Analysis Project version 2 - GLODAPv2 database [1] was used to estimate the values of $\delta^{13}C$ in the dissolved inorganic carbon ($\delta^{13}C_{DIC}$) within the study area, based on the apparent oxygen utilization (AOU) values. AOU is the difference between the measured dissolved oxygen concentration and its equilibrium saturation concentration in water with the same physical and chemical properties, and is a good indicator of oxidation of organic material, which in turn controls the distribution of $\delta^{13}C_{DIC}$ [2]. A simple linear regression was calculated to predict $\delta^{13}C_{DIC}$ based on *AOU* ($F_{(1,303)}=1.1*10^5$, $p<0.001$), with an $R^2=0.785$:

$$\delta^{13}C_{DIC} = 1.349 - 0.006\ AOU,$$

where $\delta^{13}C_{DIC}$ is measured in ‰ VPDB and *AOU* is measured in µmol kg^{-1} (Fig. S1) [2,3].

Individual tagging data and trawl surveys indicate that Icelandic cod occur mainly in the depth range 50-350 m [4,5], while NEA cod is mainly distributed in the depth range 150 -350 m [6–8]. Mean and standard deviation of $\delta^{13}C_{DIC}$ was calculated based on all predicted $\delta^{13}C_{DIC}$ values within these depths and limited geographical areas (60°N – 68°N, 30°W – 10°W for Icelandic cod and 66°N – 80°N, 5°E – 50°E for NEA cod; Fig. 1 in the main text). The mean value of predicted $\delta^{13}C_{DIC}$ within the selected areas and depth ranges equals 1.25 (SD=0.07) and 1.27 (SD=0.09) ‰ VPDB for Icelandic and NEA cod, respectively (Fig. S2, Fig. S3). These predicted $\delta^{13}C_{DIC}$ values were assumed to be representative of the carbon isotopic conditions in the waters occupied by each stock and they were used in the calculation of proportion of metabolically derived carbon in otolith carbonate (C_{resp}) based on the $\delta^{13}C$ in otoliths (Tab. 1).

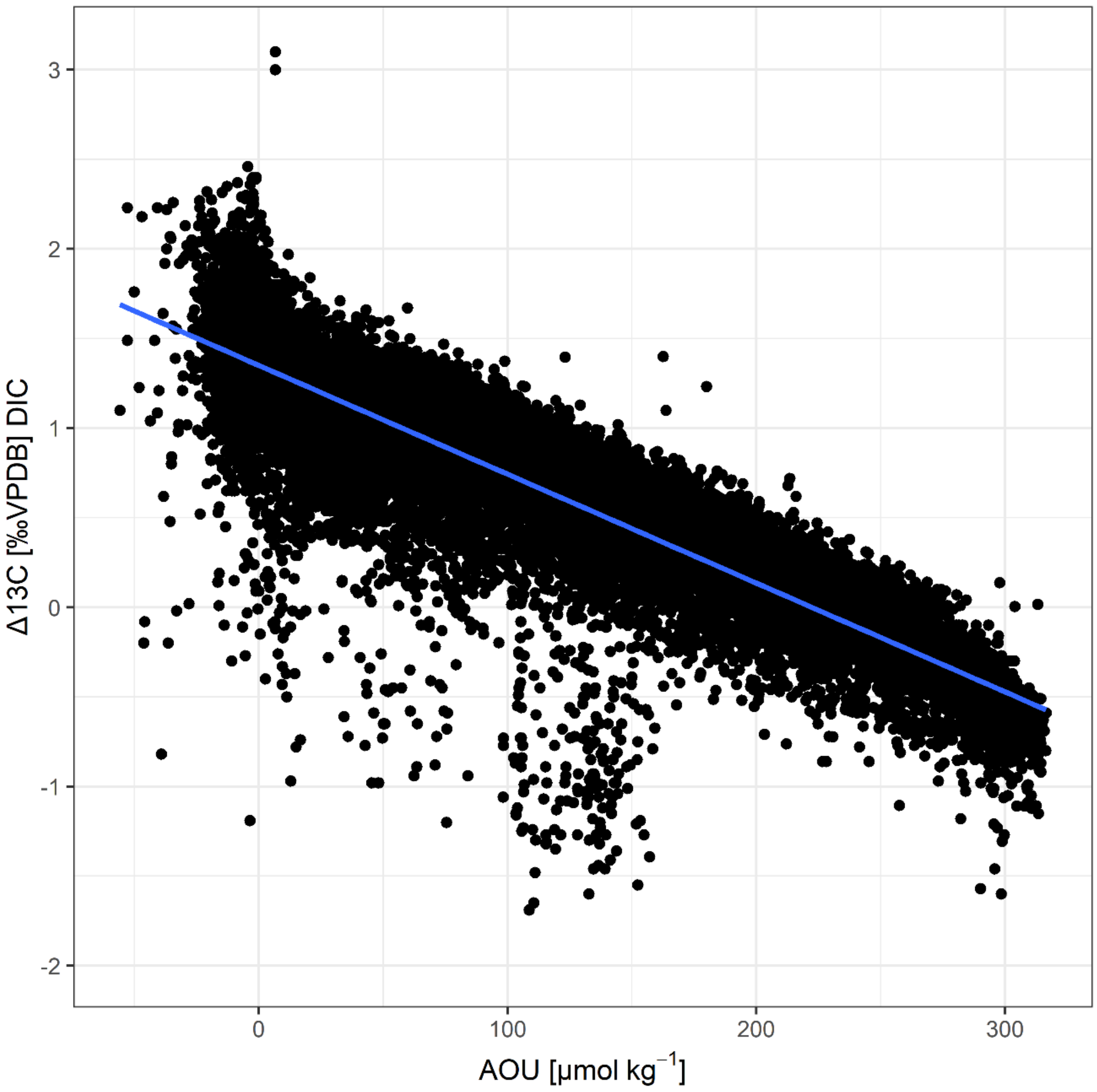

Fig. S1. Relationship between $\delta^{13}C_{DIC}$ and *AOU* based on the GLODAPv2 database. Four outlying points ($\delta^{13}C$=< -2) were excluded during visualization.

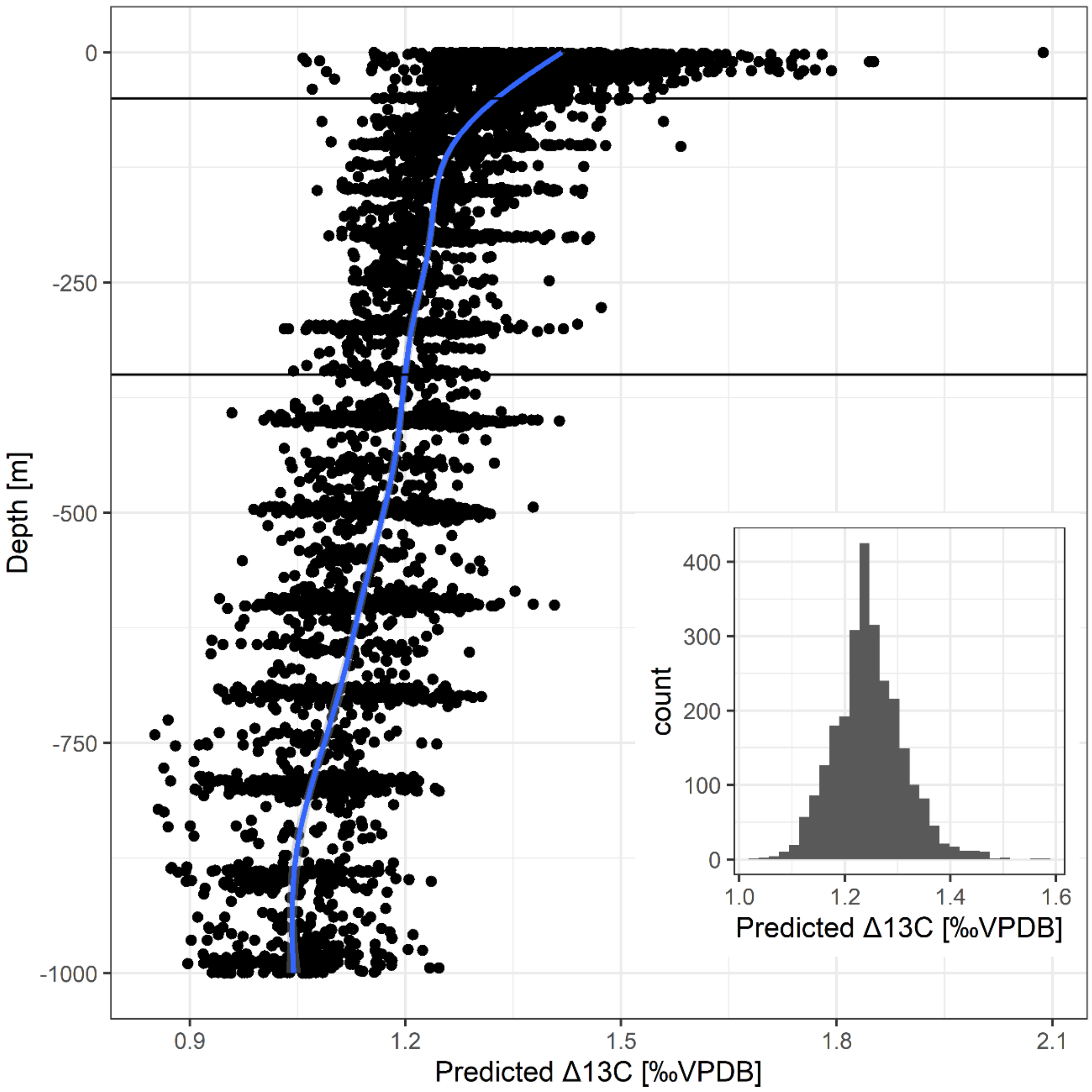

Fig. S2. Distribution of predicted $\delta^{13}C_{DIC}$ within the Icelandic cod study area (60°N – 68°N, -30°E – -10°E) as a function of depth. Depth range typical for Icelandic cod (between 50 and 350 m) is indicated with two solid vertical lines. The inset plot shows the distribution of predicted $\delta^{13}C_{DIC}$ within this depth range.

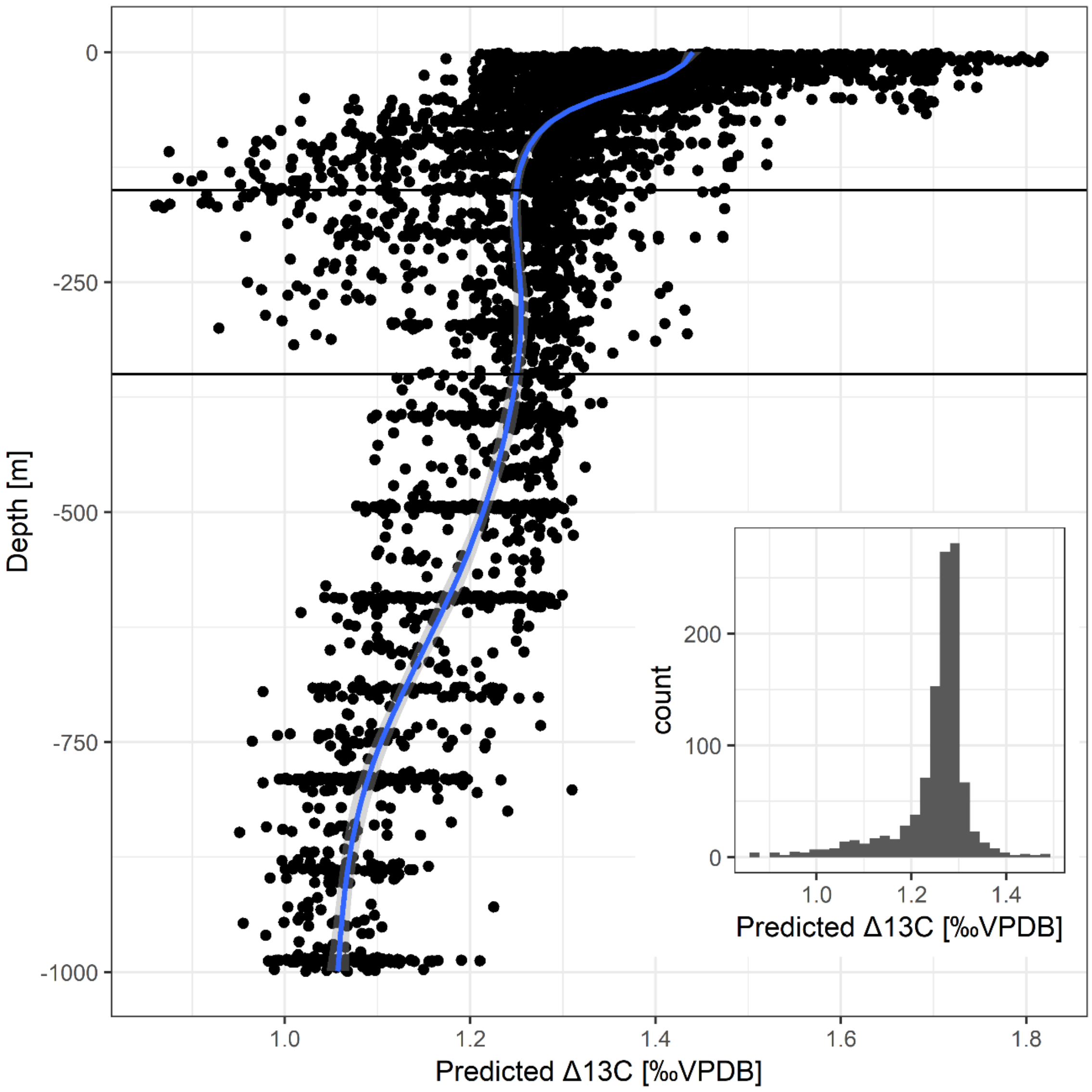

Fig. S3. Distribution of predicted $\delta^{13}C_{DIC}$ within the NEA cod study area (66°N – 80°N, 5°E – 50°E) as a function of depth. Depth range typical for NEA cod (between 150 and 350 m) is indicated with two solid vertical lines. The inset plot shows the distribution of predicted $\delta^{13}C_{DIC}$ within this depth range.

3. Approximation of $\delta^{13}C$ of cod diet

Fish, shrimp, and euphausiids dominate the diet of Icelandic cod [9]. Similarly, the NEA cod diet [10,11] is composed mostly (~80%) of fish, amphipods, krill, and shrimp. In Icelandic waters, $\delta^{13}C$ of these organisms range between -21.3 and -18.4‰ [12] and in the Barents Sea between -20.7 and -17.7‰ [13]. Considering possible differences in $\delta^{13}C_{diet}$ between life stages [9–11] the values of -19.9 ± 1.5‰ and -19.2 ± 1.5‰ were assumed in the two-component mixing model for Icelandic and NEA cod, respectively (Tab. 1 in the main text).

4. Results of the models’ selection

Table S3. Selection of the optimal random effects in the univariate model of $\delta^{13}C_{oto}$. Series of models were fitted to the data with the full intrinsic fixed-effects structure (age, total length). Based on Akaike Information Criterion corrected for the small sample sizes (AICc) the best model was selected (in bold).

Random intercept	df	AICc	ΔAICc
**FishID, StockYear**	**17**	**1368.03**	**0**
StockYear	16	1456.29	88.27
FishID	16	1398.59	30.56

Table S4. Selection of the optimal fixed intrinsic effects in the univariate model of $\delta^{13}C_{oto}$. Series of models were fitted to the data with the optimal random effects structure (Tab. S3). Based on Akaike Information Criterion corrected for the small sample sizes (AICc) the best model was selected (in bold). Parameter estimates of continuous variables are given in the selection table and “+” indicates that factor variable was included in the given model.

Intercept	Age	TL	Sex	Stock	Age: Sex	Age: Stock	TL: Stock	Sex: Stock	Age: Sex: Stock	df	AICc	ΔAICc
**-1.541**	**+**	**-0.325**		**+**		**+**				**8**	**1314.695**	**0**
-1.567	+	-0.324		+						7	1315.166	0.47
-1.542	+			+		+				7	1315.246	0.551
-1.542	+	-0.09		+		+	+			9	1315.613	0.918
-1.568	+			+						6	1315.714	1.018
-1.568	+	-0.094		+			+			8	1316.125	1.429
-1.539		-0.321		+						6	1316.43	1.735
-1.541				+						5	1316.923	2.228
-1.54		-0.09		+			+			7	1317.385	2.689
-1.478	+		+	+		+		+		11	1318.503	3.807
-1.547	+	-0.317	+	+		+				10	1318.515	3.82
-1.556	+		+	+		+				9	1318.889	4.194
-1.504	+		+	+				+		10	1318.96	4.265
-1.573	+	-0.316	+	+						9	1318.962	4.267
-1.481	+	-0.239	+	+		+		+		12	1319.243	4.547
-1.581	+		+	+						8	1319.336	4.64
-1.541	+	-0.098	+	+		+	+			11	1319.594	4.898

-1.507	+	-0.239	+	+				+		11	1319.69	4.995
-1.567	+	-0.103	+	+			+			10	1320.082	5.386
-1.476			+	+				+		9	1320.201	5.506
-1.545		-0.313	+	+						8	1320.25	5.554
-1.554			+	+						7	1320.572	5.877
-1.479	+	-0.057	+	+		+	+	+		13	1320.608	5.912
-1.479		-0.236	+	+				+		10	1320.966	6.27
-1.505	+	-0.063	+	+			+	+		12	1321.086	6.39
-1.54		-0.098	+	+			+			9	1321.357	6.661
-1.482	+		+	+	+	+		+		13	1322.238	7.543
-1.551	+	-0.315	+	+	+	+				12	1322.252	7.557
-1.586	+	-0.314	+	+	+					11	1322.281	7.585
-1.517	+		+	+	+			+		12	1322.32	7.625
-1.477		-0.058	+	+			+	+		11	1322.35	7.655
-1.56	+		+	+	+	+				11	1322.591	7.896
-1.595	+		+	+	+					10	1322.61	7.915
-1.484	+	-0.238	+	+	+	+		+		14	1323.003	8.308
-1.52	+	-0.238	+	+	+			+		13	1323.078	8.382
-1.545	+	-0.097	+	+	+	+	+			13	1323.344	8.649
-1.58	+	-0.102	+	+	+		+			12	1323.423	8.727
-1.482	+	-0.056	+	+	+	+	+	+		15	1324.38	9.684
-1.518	+	-0.062	+	+	+		+	+		14	1324.492	9.797
-1.468	+		+	+	+	+		+	+	15	1325.877	11.181
-1.471	+	-0.235	+	+	+	+		+	+	16	1326.677	11.981
-1.469	+	-0.056	+	+	+	+	+	+	+	17	1328.076	13.381
-1.819	+		+							7	1354.275	39.58
-1.815	+	-0.233	+							8	1355.072	40.377
-1.761	+									5	1355.488	40.792
-1.76	+	-0.28								6	1355.713	41.018
-1.791			+							6	1356.123	41.428
-1.787		-0.228	+							7	1356.98	42.285
-1.731										4	1357.546	42.851
-1.831	+		+		+					9	1357.615	42.919
-1.73		-0.275								5	1357.841	43.146
-1.827	+	-0.231	+		+					10	1358.453	43.757

Table S5. Selection of the optimal fixed extrinsic effects in the univariate model of $\delta^{13}C_{oto}$. Series of models were fitted to the data with the optimal random effects and fixed intrinsic effects structure (Tab. S3, Tab. S4). Based on Akaike Information Criterion corrected for the small sample sizes (AICc) the best model was selected (in bold). Parameter estimates of

continuous variables are given in the selection table and “+” indicates that factor variable was included in the given model.

Intercept	Age	AnomT	TL	Year	Stock	Age: Year	Age: Stock	AnomT: Stock	Year: Stock	Age: Year: Stock	df	AICc	ΔAICc
**-1.563**	**+**		**-0.203**	**-0.007**	**+**		**+**		**+**		**10**	**1288.898**	**0**
-1.558	+		-0.189	-0.005	+		+				9	1289.967	1.069
-1.565	+		-0.206	-0.007	+	+	+		+		11	1290.625	1.727
-1.563	+	-0.007	-0.206	-0.007	+		+		+		11	1290.936	2.038
-1.559	+		-0.192	-0.006	+	+	+				10	1291.73	2.832
-1.567	+		-0.203	-0.008	+	+	+		+	+	12	1292.001	3.103
-1.558	+	0.001	-0.189	-0.005	+		+				10	1292.015	3.117
-1.565	+	-0.009	-0.21	-0.007	+	+	+		+		12	1292.655	3.757
-1.563	+	0.021	-0.21	-0.007	+		+	+	+		12	1292.843	3.945
-1.559	+	-0.001	-0.192	-0.006	+	+	+				11	1293.783	4.885
-1.567	+	-0.007	-0.206	-0.008	+	+	+		+	+	13	1294.051	5.153
-1.557	+	0.009	-0.19	-0.005	+		+	+			11	1294.057	5.159
-1.565	+	0.02	-0.214	-0.007	+	+	+	+	+		13	1294.55	5.652
-1.559	+	0.008	-0.193	-0.006	+	+	+	+			12	1295.826	6.928
-1.567	+	0.019	-0.21	-0.008	+	+	+	+	+	+	14	1295.99	7.092
-1.541	+		-0.325		+		+				8	1314.695	25.797
-1.541	+	-0.05	-0.339		+		+				9	1315.997	27.099
-1.541	+	-0.033	-0.341		+		+	+			10	1318.001	29.103

Table S6. Comparison of the baseline model of $\delta^{13}C_{oto}$ (selected from Table S5) and extended model with “spawning zones” term (SZ) included. Models were compared based on the limited data (N=722), where information about the assignment of otolith increment to “spawning zones” (SZ: yes/no) was available.

Intercept	Age	TL	Year	Stock	Age: Stock	Year: Stock	SZ	df	AICc	ΔAICc
**-1.540**	**+**	**-0.421**	**-0.008**	**+**	**+**	**+**		**10**	**1107.159**	**0**
-1.540	+	-0.420	-0.008	+	+	+	+	11	1109.216	2.057

Table S7. Selection of the optimal random effects in the univariate model of otolith increment width. Series of models were fitted to the data with the full intrinsic fixed-effects structure (age, total length). Based on Akaike Information Criterion corrected for the small sample sizes (AICc) the best model was selected (in bold).

Model	df	AICc	ΔAICc
**FishID, StockYear**	**17**	**382.71**	**0**
Year	16	463.79	81.08
FishID	16	399.57	16.87

Table S8. Selection of the optimal fixed intrinsic effects in the univariate model of otolith increment width. Series of models were fitted to the data with the optimal random effects structure (Tab. S7). Based on Akaike Information Criterion corrected for the small sample sizes (AICc) the best model was selected (in bold). Parameter estimates of continuous variables are given in the selection table and "+" indicates that factor variable was included in the given model.

Intercept	Age	TL	Sex	Stock	Age: Sex	Age: Stock	TL: Stock	Sex: Stock	Age: Sex: Stock	df	AICc	delta
**5.146**	**-0.649**	**0.294**		**+**		**+**				**8**	**293.34**	**0**
5.146	-0.648	0.265		+		+	+			9	294.973	1.633
5.143	-0.649	0.296	+	+		+				10	296.806	3.465
5.142	-0.649	0.259	+	+		+	+			11	298.177	4.837
5.146	-0.67	0.3	+	+	+	+		+	+	16	300.075	6.735
5.146	-0.649	0.301	+	+		+		+		12	300.367	7.027
5.143	-0.655	0.296	+	+	+	+				12	300.495	7.155
5.145	-0.67	0.261	+	+	+	+	+	+	+	17	301.354	8.014
5.145	-0.649	0.262	+	+		+	+	+		13	301.647	8.306
5.142	-0.655	0.259	+	+	+	+	+			13	301.866	8.525
5.146	-0.655	0.302	+	+	+	+		+		14	304.032	10.692
5.145	-0.655	0.262	+	+	+	+	+	+		15	305.306	11.966
5.146	-0.645			+		+				7	335.117	41.777
5.151	-0.646		+	+		+				9	338.517	45.177
5.142	-0.667		+	+	+	+		+	+	15	340.631	47.29
5.142	-0.646		+	+		+		+		11	341.219	47.879
5.151	-0.652		+	+	+	+				11	342.204	48.864
5.142	-0.652		+	+	+	+		+		13	344.944	51.604
5.143	-0.56	0.292	+	+	+					11	374.698	81.358
5.141	-0.56	0.241	+	+	+		+			12	375.511	82.171
5.145	-0.583	0.289		+						7	377.98	84.64
5.145	-0.56	0.296	+	+	+			+		13	378.447	85.107
5.145	-0.583	0.242		+			+			8	378.992	85.652
5.144	-0.56	0.244	+	+	+		+	+		14	379.157	85.817
5.142	-0.583	0.291	+	+						9	381.726	88.386
5.141	-0.583	0.237	+	+			+			10	382.404	89.064

5.169	-0.56	0.282	+		+			10	382.683	89.343
5.145	-0.583	0.297	+	+			+	11	385.359	92.019
5.144	-0.583	0.241	+	+		+	+	12	385.905	92.565
5.165	-0.583	0.285						6	387.297	93.957
5.169	-0.583	0.281	+					8	389.84	96.5
5.151	-0.558		+	+	+			10	414.441	121.101
5.141	-0.558		+	+	+		+	12	417.051	123.711
5.145	-0.58			+				6	417.64	124.3
5.173	-0.558		+		+			9	419.118	125.777
5.15	-0.58		+	+				8	421.215	127.874
5.141	-0.58		+	+			+	10	423.992	130.652
5.164	-0.581							5	424.852	131.512
5.173	-0.581		+					7	425.983	132.643
5.131		0.011		+		+		7	4285.705	3992.365
5.131		0.118		+				6	4286.74	3993.4
5.131				+				5	4288.774	3995.434
5.125		0.011	+	+		+		9	4289.229	3995.889
5.128		0.119	+	+				8	4290.319	3996.979
5.127		0.017	+	+		+	+	11	4290.931	3997.591
5.129		0.127	+	+			+	10	4292.282	3998.942
5.131			+	+				7	4292.377	3999.037
5.127			+	+			+	9	4294.669	4001.328
5.165		0.113						5	4297.913	4004.573
5.165								4	4299.48	4006.139
5.171		0.107	+					7	4301.296	4007.956
5.173			+					6	4302.489	4009.149

Table S9. Selection of the optimal fixed extrinsic effects in the univariate model of otolith increment width. Series of models were fitted to the data with the optimal random effects and fixed intrinsic effects structure (Tab. S7, Tab. S8). Based on Akaike Information Criterion corrected for the small sample sizes (AICc) the best model was selected (in bold). Parameter estimates of continuous variables are given in the selection table and "+" indicates that factor variable was included in the given model.

Intercept	AnomalyTemp	Age	TL	Year	Stock	AnomalyTemp: Stock	Age: Stock	Year: Stock	df	AICc	delta
**5.146**	**0.02**	**-0.649**	**0.301**		**+**		**+**		**9**	**292.653**	**0**
5.146		-0.649	0.294		+		+		8	293.34	0.687
5.146	0.021	-0.649	0.305	0	+		+		10	294.391	1.738
5.146	0.017	-0.649	0.302		+	+	+		10	294.621	1.968
5.146		-0.649	0.295	0	+		+		9	295.285	2.632
5.146	0.017	-0.649	0.305	0	+	+	+		11	296.357	3.703

5.146	0.021	-0.649	0.305	0	+		+	+	11	296.386	3.733
5.146		-0.649	0.295	0	+		+	+	10	297.295	4.641
5.146	0.017	-0.649	0.306	0	+	+	+	+	12	298.343	5.69

References:

1. Olsen A, Kozyr A, Lauvset SK, Hoppema M, Pérez FF, Steinfeldt R, et al. The Global Ocean Data Analysis Project version 2 (GLODAPv2) – an internally consistent data product for the world ocean. Earth Syst Sci Data. 2016;8: 297–323. doi:10.5194/essd-8-297-2016

2. Kroopnick PM. The distribution of 13C of ∑CO2 in the world oceans. Deep Res. 1985;32: 57–84.

3. Filipsson HL, McCorkle DC, Mackensen A, Bernhard JM, Andersson LS, Naustvoll LJ, et al. Seasonal variability of stable carbon isotopes (δ13CDIC) in the Skagerrak and the Baltic Sea: Distinguishing between mixing and biological productivity. Palaeogeogr Palaeoclimatol Palaeoecol. 2017;483: 15–30. doi:10.1016/j.palaeo.2016.11.031

4. Pálsson ÓK, Thorsteinsson V. Migration patterns, ambient temperature, and growth of Icelandic cod (*Gadus morhua*): evidence from storage tag data. Can J Fish Aquat Sci. 2003;60: 1409–1423. doi:10.1139/f03-117

5. Begg GA, Marteinsdottir G. Environmental and stock effects on spatial distribution and abundance of mature cod *Gadus morhua*. Mar Ecol Prog Ser. 2002;229: 245–262. doi:10.3354/meps229245

6. Stensholt BK. Cod migration patterns in relation to temperature: Analysis of storage tag data. ICES J Mar Sci. 2001;58: 770–793. doi:10.1006/jmsc.2001.1067

7. Ottersen G, Michalsen K, Nakken O. Ambient temperature and distribution of north-east Arctic cod. ICES J Mar Sci. 1998;55: 67–85. doi:10.1006/jmsc.1997.0232

8. Strand KO, Sundby S, Albretsen J, Vikebø FB. The northeast Greenland shelf as a

potential habitat for the Northeast Arctic cod. Front Mar Sci. 2017;4: 1–14. doi:10.3389/fmars.2017.00304

9. Pálsson ÓK, Bjrnsson H. Long-term changes in trophic patterns of Iceland cod and linkages to main prey stock sizes. ICES J Mar Sci. 2011;68: 1488–1499. doi:10.1093/icesjms/fsr057

10. Bogstad B, Haug T, Mehl S. Who eats whom in the Barents Sea? NAMMCO Sci Publ. 2000;2: 98–119. doi:10.7557/3.2975

11. Holt RE, Bogstad B, Durant M. Barents Sea cod (*Gadus morhua*) diet composition: long-term interannual, seasonal, and ontogenetic patterns. ICES J Mar Sci. 2019;76: 1641–1652. doi:10.1093/icesjms/fsz027

12. Thompson DR, Lilliendahl K, Solmundsson J, Furness RW, Waldron S, Phillips RA. Trophic relationships among six species of Icelandic seabirds as determined through stable isotope analysis. Condor. 1999;101: 898–903.

13. Tamelander T, Renaud PE, Hop H, Carroll ML, Ambrose WG, Hobson KA. Trophic relationships and pelagic-benthic coupling during summer in the Barents Sea Marginal Ice Zone, revealed by stable carbon and nitrogen isotope measurements. Mar Ecol Prog Ser. 2006;310: 33–46. doi:10.3354/meps310033
